# Prevalence, Awareness, Treatment, Control and Risk Factors Associated with Hypertension among Adults in Southern China, 2013

**DOI:** 10.1371/journal.pone.0146181

**Published:** 2016-01-19

**Authors:** Li Yang, Jing Yan, Xinhua Tang, Xiaoling Xu, Wei Yu, Haibin Wu

**Affiliations:** 1 Zhejiang Provincial Center for Cardiovascular Disease Control and Prevention, Zhejiang hospital, Hangzhou, China; 2 Zhejiang Provincial Center for Disease Control and Prevention, Hangzhou, China; Shanghai Institute of Hypertension, CHINA

## Abstract

To investigate the prevalence, awareness, treatment, control of hypertension and their associated factors in southern China. A cross-sectional survey was conducted in 5 cities of urban areas and 5 counties of rural areas in Southern China in 2013, a stratified multistage random sampling method was used to select a representative sample. Recruitment included a total of 19254 participants aged 15 or older. Socio-demographic profiles, examinations were administrated on each subject. Multilevel logistic regression models were used to identify the risk factors of hypertension, awareness, treatment, and control. Overall, the prevalence of hypertension and pre-hypertension are 24.59% and 32.11%, respectively in southern China. Among all the hypertensive patients, 67.43% were aware of their condition, 55.76% took anti-hypertension medication recent two weeks, and 30.79% had their blood pressure controlled. Compared with male, female hypertensive patients had higher rates of hypertension awareness, treatment and control. Age, gender, marital status, living areas, education, BMI, waist circumference, visceral adipose index (VAI), high body fat percentage (BFP) and family hypertension history correlated with the prevalence of hypertension. SBP/DBP increased with VAI and BFP increasing. There is an increasing prevalence of hypertension and high pre-hypertension in the general population in southern China, but levels of awareness, treatment, and control remain relatively low, especially for young and middle-aged population. Innovative strategies including of adopting appropriate anti-hypertensive medication therapy and healthy lifestyles should be taken.

## Introduction

Cardiovascular disease (CVD) is one of the leading causes of death and burden worldwide, hypertension has ranked first as a modifiable risk factor of CVD in China [1]. According to the 2002 National Nutrition and Health Survey (NNHS), the prevalence of hypertension in China among adults 18 years or more was 18.8%, the awareness, treatment and control of hypertension were only 30%, 25% and 6%, respectively [2].

Zhejiang province located in Southern China has a population of approximately 55 million in 2013. The result from NNHS in 2002 showed that the prevalence of hypertension among adults 18 years or more in Zhejiang province was 19.8%, the rates of awareness, treatment and control among hypertensive were 37.13%, 29.61% and 10.15%, respectively [2, 3]. As one of the most rapidly developing provinces, dramatic social and economic changes, including rapid urbanization has occurred in Zhejiang over the past 2 decades. Since the survey in 2002, there were no large-scale surveys on hypertension in Zhejiang province, we hope to ascertain the current status of hypertension in this area [1, 4].

## Methods

### Ethics statement

Ethics approval was obtained from the Zhejiang Hospital Ethics Review Board and the Fuwai Hospital Ethics Review Board both. Written informed consent was obtained from each participant. The ethics committee approved this procedure.

### Sampling

We used stratified multistage random sampling method to select representative samples. On the basis of administrative data, it was divided into urban areas and rural areas. Using the probability proportional to size (PPS) method, 5 cities in urban areas and 5 counties in rural areas were selected. We chose two districts or two townships within each city and county, and three communities or villages within each district and township respectively using the simple random sampling (SRS) method. Finally, a given number of participants from each of the 14 gender/age strata (male/female and aged 15–24, 25–34, 35–44, 45–54, 55–64, 65–74, ≥ 75) were chosen also using the SRS method according to the national demographic composition, from communities or villages using the lists compiled from the local government registers of households[4].

As a result of the complex sample, we also considered the design effect when we estimated the sample size. Assuming a design effect of 2.5 and the prevalence of hypertension among population aged 15 years or more of 17.7%, 19000 participants are needed to ensure that the average lengths of the 95% confidence interval (CI) for the prevalence in the entire population and subpopulation defined by age and gender are less than 0.4% and 1.8%, respectively. According to the design, a total of 28000 participants were randomly selected from 10 urban cities and rural counties and invited to participate in the study.

### Measurement

Data collection included a questionnaire interview, physical examinations and biochemical examinations. We used the standardized questionnaire developed by the national coordinating center, Fuwai Hospital (Beijing, China), which was administered by trained general practitioners during a face-to-face individual interview. It included demographic information such as age, education and health behaviors such as history of smoking, alcohol consumption, diet and physical activity.

We measured blood pressure (BP) with the OMRON HBP-1300 Professional Portable Blood Pressure Monitor (OMRON, Kyoto, Japan) three times on the right arm supported at heart level after the participant sitting at rest for 5 min, with 30 s between each measurement. The average of the three readings would be used for analysis [5,6]. Height was measured without shoes using a standard right angle device and a fixed measurement tape (to the nearest 0.5 cm), and waist circumference in a standing position using a cloth tape directly on the participant's skin (to the nearest 0.5 cm). Body weight without heavy clothing, as well as body fat, visceral fat, was measured using an OMRON body fat and weight measurement device (V-body HBF-371, OMRON, Kyoto, Japan).

### Definition

Hypertension is defined as SBP ≥ 140 mmHg and/or DBP ≥ 90 mmHg, or self-reported treatment of hypertension with antihypertensive medication. Pre-hypertension is defined as 120 mmHg ≤ SBP ≤ 139 mmHg and/or 80 mmHg ≤ DBP ≤ 89 mmHg, a BP goal of less than 140/90 mmHg for hypertensive is defined as control of hypertension [7,8]. Awareness of hypertension was defined as self-report of any previous diagnosis of hypertension by a healthcare professional before the study. Treatment of hypertension was defined as self-reported use of a prescription medication for management of hypertension during the previous 2 weeks [7–9].

Overweight is defined as body mass index (BMI) ≥ 24 kg/m^2^ and < 28 kg/m^2^, obesity was defined as BMI ≥ 28kg/m^2^ [10]. VAI is classified as standard, slightly high and high [11]. Fasting plasma glucose (FPG) is classified as impaired fasting glucose with FBG ≥ 6.1 mmol/L and ≤ 6.99 mmol/L, diabetes mellitus (DM) as FBG ≥ 7.0 mmol/L[10]. Serum triglycerides (TG) is high with TG ≥ 2.26 mmol/L, serum total cholesterol (TC) is high with TC ≥ 6.22 mmol/L. Low serum high-density lipoprotein cholesterol (HDL-C) are defined as HDL-C < 1.04 mmol/L[10].

Subjects who smoked one cigarette or more per day for over 6 months were defined as smokers, and alcohol drinkers were assessed by asking subjects whether they had consumed more than once every week in the last 12 months. Subjects who consumed more than 6 grams salt per day for over 6 months were defined as excessive salt users [12]. We assessed salt consumption and fruit intake by using a retrospective method of dietary assessment from the food frequency questionnaire designed by Fuwai Hospital (Beijing).

### Statistical analysis

The sampling design including stratification, clustering and sampling weights was taken into account in all estimates and analyses using the specific SAS commands. Epidata 3.0 was used for data entry and validation and SAS 9.2 for data management and analysis. Frequencies (percentages) or means and standard deviations were used to summarize the socio-demographic characteristics, physical measurements and hypertension status of participants. Continuous variables and categorical variables were compared by the Student’s t-test/variance analysis and Chi-square test, respectively. The trends in prevalence of hypertension associated factors across categories were analyzed using Chi-square test. And the strength of associations of socio-demographic associated factors of hypertension, awareness, treatment and control were assessed by Odds-Ratios (OR) estimated in logistic regression models. Crude associations were first assessed using univariate models, then associations were assessed using multivariate models adjusted for the covariates age, gender, region, education, retired status, marital status, BMI and family history of hypertension. Significance level was set at p<0.05 for all hypothesis tests.

## Results

A total of 19254 study participants from 28000 eligible participants completed all of the questionnaires, measurement examinations. The sex ratio was similar between responders and nonresponders (*P*>0.05). However, nonresponders were significantly younger than the responders, probably because of their work habits (35 ± 16 years vs. 46 ± 19 years; *P* < 0.05).

### Prevalence and distribution of hypertension

Table 1 showed the prevalence and distribution of normotension, pre-hypertension and hypertension by demographic and socio-economic status among the adults. The prevalence of hypertension and pre-hypertension were 24.59% and 32.11%, respectively. The prevalence of hypertension increased with age and decreased with education levels. The prevalence of the normotension, the pre-hypertension and hypertension was different among the BMI categories and waist circumference categories (*P*<0.0001, Table 1). The hypertensive had higher BMI, waist circumference, body fat percentage and visceral adipose index, compared with participants having normotension and pre-hypertension (Table 1).

**Table 1:** Distribution of hypertension by socio demographic and lifestyle factors among the adults of Southern China in 2013 (n = 19254).

Characteristics	Normotension (N = 8336)	Pre-hypertension (N = 6183)	Hypertension (N = 4735)	P-value
Prevalence, n (%)	8336 (43.3)	6183 (32.1)	4735 (24.6)	<0.0001
Age, 15–39, n (%)	5257 (63.5)	2679 (32.4)	325 (3.9)	<0.0001
40–49, n (%)	1483 (44.6)	1270 (38.2)	571 (17.2)	
50–59, n (%)	777 (31.4)	846 (34.24)	848 (34.3)	
60 and above, n (%)	807 (15.6)	1383 (26.72)	2985 (57.7)	
Mean ± SD	36.0 ± 15.7	44.5 ± 17.9	63.1 ± 15.0	<0.0001
Sex men, n (%)	3262 (34.1)	3839 (40.1)	2480 (25.9)	<0.0001
Area of residence, n (%)				
Urban	4223 (43.2)	3202 (32.8)	2352 (24.1)	0.0871
Rural	4113 (43.4)	2981 (31.5)	2383 (25.2)	
Education, n (%)				
Illiterate	680 (20.6)	956 (28.9)	1672 (50.5)	<0.0001
Primary	1265 (31.1)	1258 (31.0)	1540 (37.9)	
Middle	4945 (53.7)	2953 (32.1)	1314 (14.3)	
College or higher	1445 (54.1)	1015 (38.0)	209 (7.8)	
Family history of hypertension, n (%)	2481 (30.3)	1975 (32.9)	1510 (34.5)	<0.0001
Smoker, n (%)	1339 (32.4)	1660 (40.2)	1136 (27.5)	<0.0001
Alcohol intake, n (%)	1541 (31.0)	1857 (37.4)	1570 (31.6)	<0.0001
Excessive salt, n (%)	3363 (42.1)	2447 (30.7)	2172 (27.2)	<0.0001
Adequate physical activity, n (%)	6158 (43.5)	4481 (31.6)	3528 (24.9)	0.0081
Fruit intake (g / day), Mean (SD)	174.5 ± 170.8	164.1 ± 190.2	121.0 ± 167.8	<0.0001
BMI(kg/m2)				
Underweight (BMI<18.5)	1150 (65.8)	320 (18.3)	277 (15.9)	<0.0001
Normal (18.5≤BMI<24.0)	5540 (50.4)	3470 (31.6)	1973 (18.0)	
Overweight (24≤BMI<28.0)	1424 (28.2)	1843 (36.5)	1785 (35.3)	
Obese (BMI≥28.0)	222 (15.1)	550 (37.4)	700 (47.6)	
Mean ± SD	22.2 ± 31.5	23.9 ± 28.8	24.7 ± 10.0	<0.0001
AWC (cm)				
<90 for M, <85 for F, n (%)	7495 (50.2)	4683 (31.4)	2741 (18.4)	<0.0001
≥90 for M, ≥85 for F, n (%)	502 (23.6)	755 (35.5)	872 (41.0)	
≥95 for M, ≥90 for F, n (%)	338 (15.3)	745 (33.8)	1122 (50.9)	
Mean ± SD	75.8 ± 26.8	82.2 ± 21.9	86.2 ± 28.6	<0.0001
Blood pressure (mmHg)				
Systolic	109.0 ± 7.3	127.1 ± 5.7	145.2 ± 23.6	<0.0001
Diastolic	66.1 ± 6.6	75.4 ± 7.2	81.6 ± 11.7	<0.0001
Biochemical measurements (mmol/L)				
FBG	5.3 ± 1.2	5.4 ± 1.1	5.7 ± 1.4	<0.0001
TG	1.2 ± 1.1	1.7 ± 1.7	1.7 ± 1.4	<0.0001
TC	4.9 ± 0.9	5.2 ± 1.0	5.2 ± 1.1	<0.0001
HDL-C	1.4 ± 0.3	1.4 ± 0.4	1.4 ± 0.3	<0.0001
BFP				
<10 for M, <20 for F, n (%)	733 (72.2)	244 (24.0)	38 (3.7)	<0.0001
10–19 for M, 20–29 for F, n (%)	4712 (62.1)	2159 (28.4)	720 (9.5)	
20–24 for M, 30–34 for F, n (%)	2041 (35.5)	2101 (36.5)	1610 (28.0)	
≥25 for M, ≥35 for F, n (%)	753 (16.4)	1596 (35.1)	2198 (48.5)	
Mean ± SD for M	18.8 ± 6.7	21.9 ± 6.3	24.8 ± 6.0	<0.0001
Mean ± SD for F	26.7 ± 5.4	29.9 ± 6.2	34.1 ± 5.2	<0.0001
VAI				
1–9	6748 (51.6)	4033 (30.8)	2303 (17.6)	<0.0001
10–14	716 (19.7)	1387 (38.1)	1536 (42.2)	
15–30	129 (10.1)	450 (35.1)	703 (54.8)	
Mean ± SD for M	6.7 ± 5.4	9.1 ± 5.0	11.4 ± 5.6	<0.0001
Mean ± SD for F	4.5 ± 4.5	6.6 ± 4.4	8.3 ± 4.9	<0.0001

BMI: Body mass index. TG: Triglycerides. TC: Total cholesterol. HDL-C: high density lipoprotein cholesterol. AWC: Abdominal waist circumference. FBG: Fasting blood glucose. BFP: Body fat percentage. VAI: Visceral adipose index. M: Male. F: Female.

**Fig 1:**
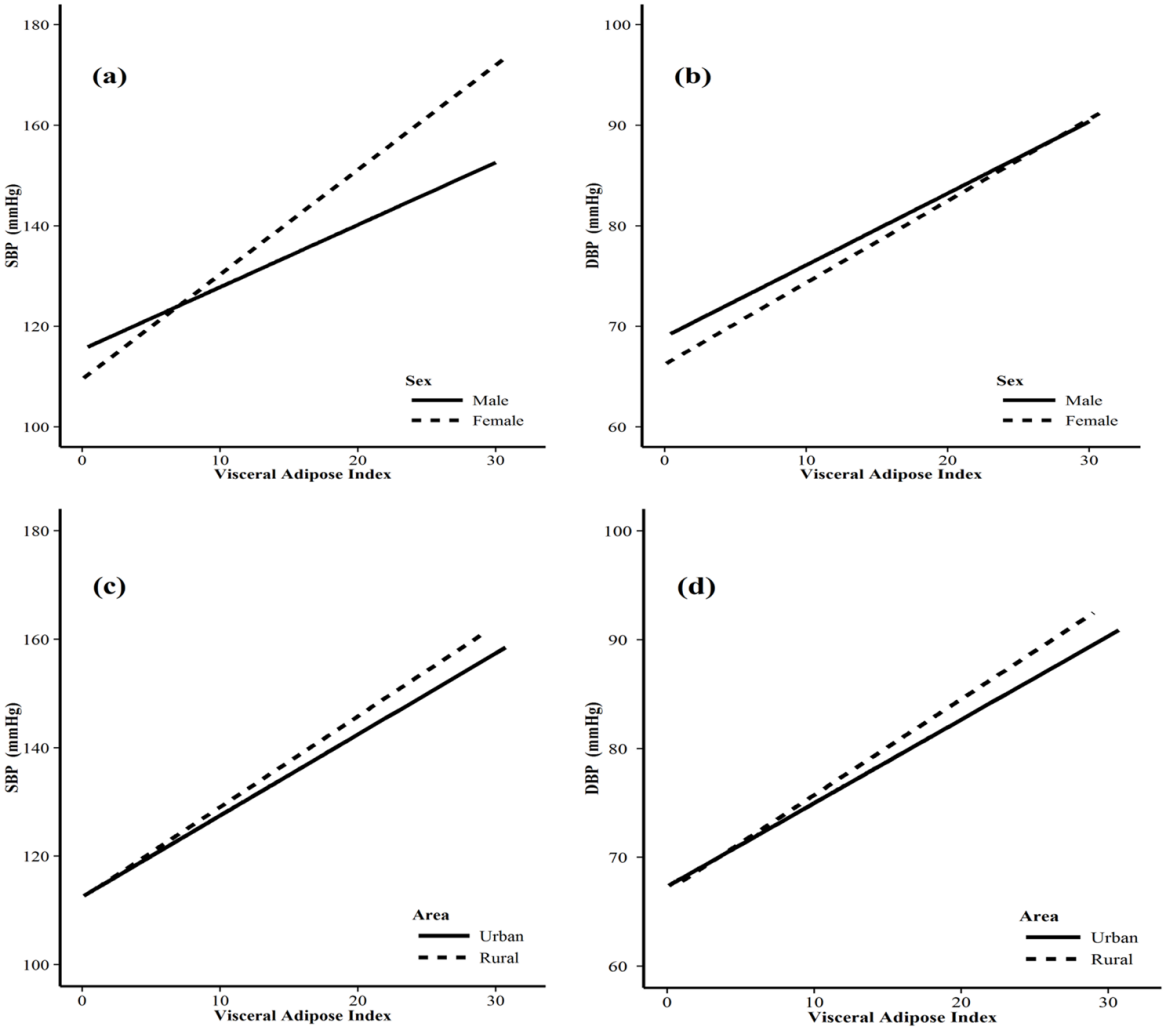
Mean systolic and diastolic blood pressure by VAI between different gender and areas. (a) mean SBP by VAI between different genders; (b) mean DBP by VAI between different genders; (c) mean SBP by VAI between different areas; (b) mean DBP by VAI between different areas.

**Fig 2:**
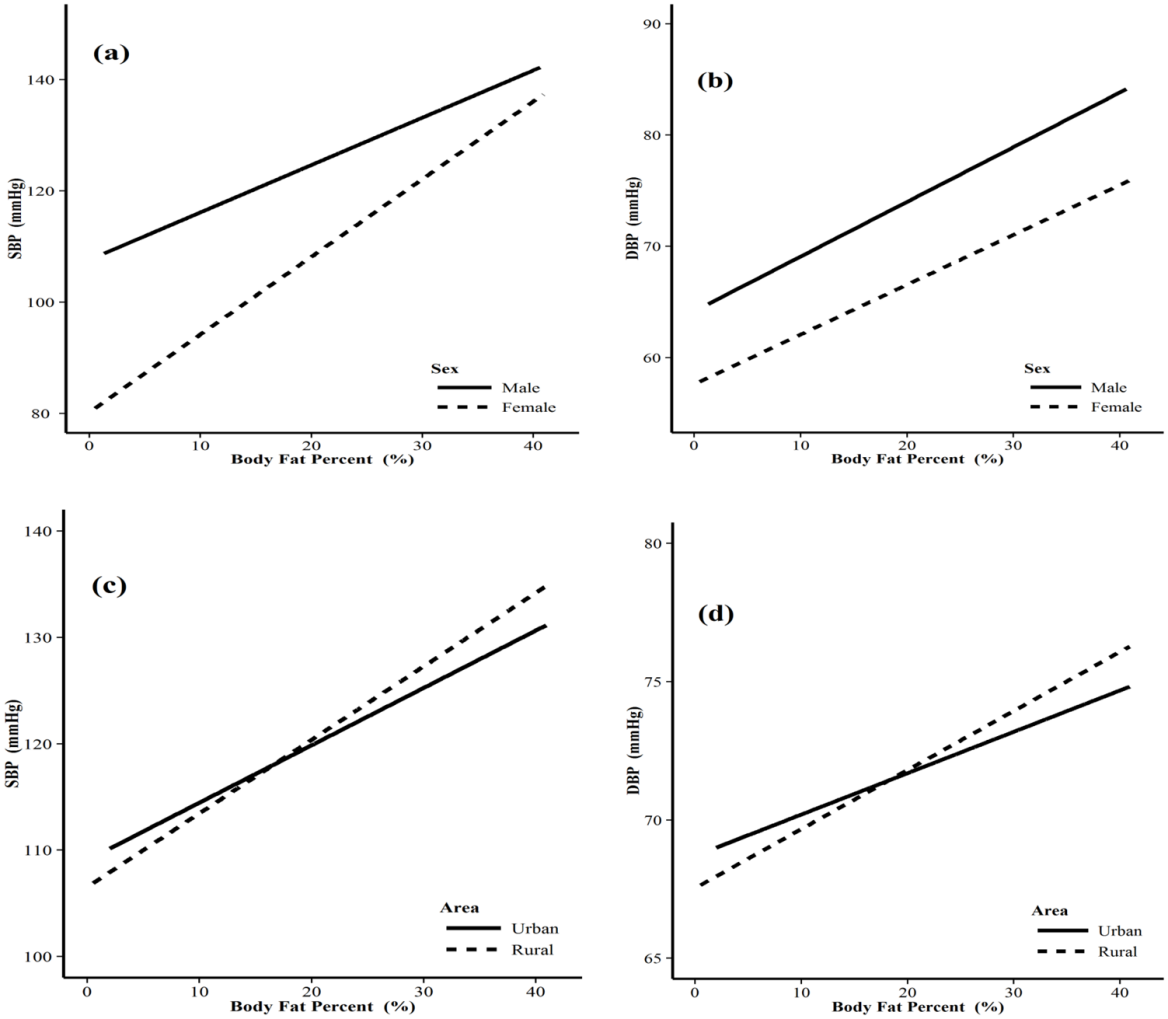
Mean systolic and diastolic blood pressure by BFP between different gender and areas. (a) mean SBP by BFP between different genders; (b) mean DBP by BFP between different genders; (c) mean SBP by BFP between different areas; (b) mean DBP by BFP between different areas.

Figs 1 and 2 showed the SBP/DBP by sex and areas how it changed with increasing visceral adipose index (VAI) and body fat percentage (BFP), respectively. The regression coefficients (RC) and 95% CI of VAI and SBP among men and women were 1.24 (1.17–1.31) and 2.09 (1.99–2.18), respectively, and the linear trend test showed that there was statistical significance (F = 211.05, P<0.0001), meaning VAI maybe had a more important effect on SBP for women, compared with men (Fig 1a). There were similar results for VAI on DBP (F = 11.42, P = 0.001) between male (RC = 0.69, 95% CI: 0.65–0.74) and female (RC = 0.81, 95% CI: 0.76–0.86) (Fig 1b). We could also find that VAI might have a more important effect on SBP (F = 10.87, P = 0.001) and DBP (F = 11.95, P = 0.001) for rural areas, compared with urban areas (Fig 1c and 1d)). Meanwhile, BFP had a more important effect on SBP (F = 193.97, P<0.0001) for female (RC = 1.39, 95% CI: 1.33–1.45), compared with male (RC = 0.85, 95% CI: 0.80–0.90) (Fig 2a), but there was no statistical difference for BFP on DBP between the two groups (F = 2.03, P = 0.154) (Fig 2c). And BFP might have a more important effect on SBP (F = 18.5, P<0.0001) and DBP (F = 8.62, P = 0.003) for rural areas, compared with urban areas (Fig 2c and 2d).

### Awareness, treatment and control of hypertension

As showed in Table 2, of the 4735 hypertensive individuals, 3161 (1540 men and 1651 women) were aware of their condition. The awareness rate of hypertension was 67.43%, there was significant difference between men and women (62.15% vs 73.25%, *P*<0.0001). Subjects living in rural places, retired, single had lower awareness rates. The treatment rate of hypertension was 55.76%, 2640 hypertensive individuals took prescription medication recent two weeks, women had a higher treatment rate than men (62.17% vs 49.92%, P<0.0001). But only 30.79% had their BP under control among hypertensive. There was no significant association between men and women (29.88% vs 31.80%, *P*>0.05). The rate of controlled hypertension was decreased with BMI, increased with age and the level of education. Smokers, drinkers, and the groups with family history of hypertension, living in rural places, retired, single, high FBG, TG or TC had lower control rate of hypertension (Table 2).

**Table 2:** Percentage of awareness, treatment and control of hypertension among hypertensives in Southern China in 2013 (N = 4735).

Demographic factors	Awareness		Treatment		Control	
	N (%)	95%CI	N (%)	95%CI	N (%)	95%CI
Percentage	3191(67.43)	66.08–68.77	2640(55.76)	54.33–57.18	1458(30.79)	29.48–32.13
Age groups, y						
15–39	66 (20.31)	16.07–25.10	28(8.62)	5.80–12.21	19(5.85)	3.56–8.98
40–49	311 (54.47)	50.28–58.61	225(39.40)	35.37–43.55	142(24.87)	21.37–28.63
50–59	573 (67.57)	64.30–70.71	454(53.54)	50.11–56.94	280(33.02)	29.86–36.30
60 and above	2237(75.02)	73.42–76.56	1930(64.66)	62.91–66.37	1015(34.00)	32.30–35.73
Male	1540(62.15)	60.20–64.06	1238(49.92)	47.93–51.91	741(29.88)	28.08–31.72
Area of residence						
Urban	1650(70.18)	68.29–72.03	1369(58.21)	56.18–60.21	833(35.42)	33.48–37.39
Rural	1541(64.72)	62.76–66.64	1271(53.34)	51.31–55.35	625(26.23)	24.47–28.04
Retired	1311(59.92)	57.83–61.98	1052(48.06)	45.95–50.18	618(28.23)	26.35–30.17
Married	2565(67.84)	66.32–69.33	2110(55.76)	54.18–57.37	1219(32.22)	30.73–33.74
Education level						
Illiterate	1196(71.57)	69.34–73.73	984(58.85)	56.45–61.22	449(26.85)	24.74–29.05
Primary	1075(69.90)	67.60–72.19	897(58.25)	55.74–60.72	521(33.83)	31.47–36.26
Middle	789(60.05)	57.34–62.71	641(48.78)	46.05–51.52	401(30.52)	28.04–33.09
College or higher	131(62.68)	55.74–69.25	118(56.46)	49.45–63.29	87(41.63)	34.87–48.63
BMI, Normal	64.00(1262)	61.83–66.12	52.71(1040)	50.48–54.93	31.02(612)	28.98–33.11
Overweight	71.30(1272)	69.14–73.39	59.55(1063)	57.23–61.84	31.88(569)	29.72–34.10
Obese	68.29(478)	64.70–71.72	55.43(388)	51.66–59.15	37.43(262)	34.15–40.90
AWC,<90 for M, <85 for F, n (%)	64.15(1757)	62.32–65.95	53.01(1453)	51.12–54.69	30.61(839)	28.89–32.37
≥90 for M, ≥85 for F, n (%)	71.10(620)	67.97–74.09	58.49(510)	55.13–61.76	33.26(290)	30.13–36.49
≥95 for M, ≥90 for F, n (%)	72.61(814)	69.90–75.21	60.34(677)	57.41–63.22	23.32(329)	26.67–32.06
Smoker	58.15(660)	55.22–61.04	44.37(504)	41.45–47.31	25.44(289)	22.93–28.08
Drinker	62.56(981)	60.11–64.97	48.92(768)	46.42–51.42	27.64(434)	25.44–29.93
Adequate physical activity	68.34(2409)	66.78–69.87	56.60(1997)	54.95–58.25	31.77(1121)	30.24–33.34
Salt use (≥6 gram)	67.33(1461)	65.31–69.30	55.29(1201)	53.17–57.40	30.29(658)	28.37–32.26
Diabetes	73.71(129)	66.54–80.07	61.14(107)	53.50–68.41	29.71(52)	23.05–37.08
TG ≥2.26	123(59.71)	52.67–66.47	95(46.12)	39.17–53.18	39(18.93)	13.82–24.96
TC ≥6.22	112(66.27)	58.61–73.35	80(47.34)	39.62–55.15	32(18.93)	13.33–25.67
HDL-C <1.04	2651(68.43)	66.94–69.89	2245(57.91)	56.33–59.47	1198(30.92)	29.59–32.57
FBG,≤6	3011(67.32)	65.92–68.69	2499(55.83)	54.36–57.29	1388(31.01)	29.66–32.39
6.1–6.9	105(71.43)	63.40–78.57	82(55.78)	47.37–63.96	44(29.93)	22.66–38.03
≥7	75(66.96)	57.44–75.56	59(52.68)	43.02–62.19	26(23.21)	15.76–32.14
BFP, <10 for M, <20 for F, n (%)	24(63.16)	45.99–78.19	21(55.26)	38.30–71.38	14(36.84)	21.81–54.01
10–19 for M, 20–29 for F, n (%)	410(57.02)	53.31–60.66	319(44.31)	40.64–48.02	231(32.09)	30.14–34.10
20–24 for M, 30–34 for F, n (%)	1021(63.42)	61.01–65.77	841(52.24)	49.76–54.70	477(29.63)	27.40–31.92
≥25 for M, ≥35 for F, n (%)	1601(73.54)	71.63–75.36	1344(61.71)	59.49–63.60	663(30.42)	27.07–33.92
VAI, 1–9	1496(64.99)	63.00–66.94	1240(53.84)	51.78–55.89	725(31.48)	29.59–33.42
10–14	1047(68.16)	65.77–70.49	857(55.79)	53.27–58.30	471(30.66)	28.36–33.04
15–30	524(74.64)	71.25–77.82	437(62.16)	58.46–65.76	215(30.58)	27.19–34.14

BMI: Body mass index. TG: Triglycerides. TC: Total cholesterol. HDL-C: high density lipoprotein cholesterol. FBG: Fasting blood glucose. BFP: Body fat percentage. VAI: Visceral adipose index. M: Male. F: Female. AWC: Abdominal waist circumference.

### Antihypertensive medications among treated patients

**Fig 3:**
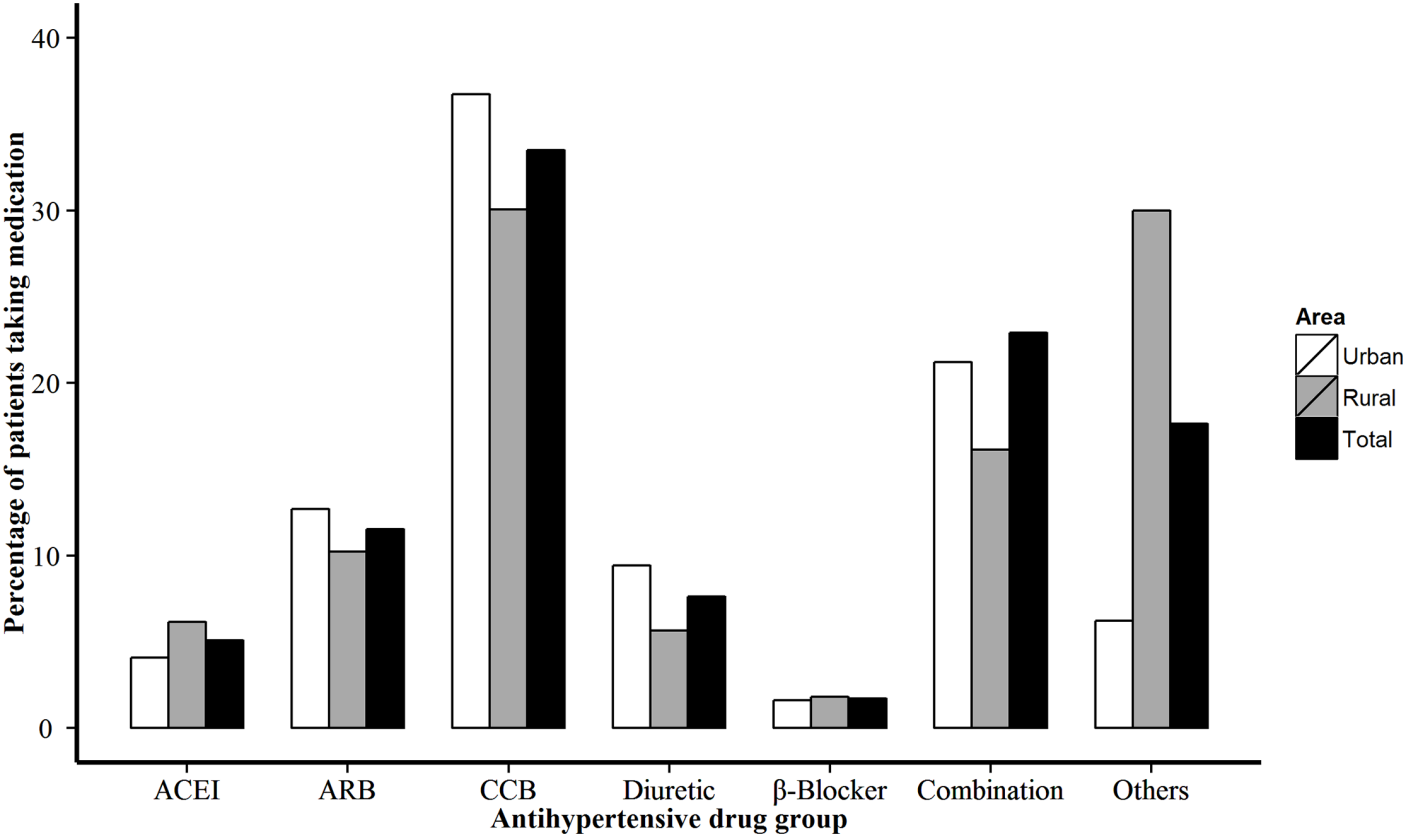
Distribution of antihypertensive medications prescribed. ACEI = angiotensin—converting enzyme inhibitors, ARB = angiotensin—receptor blocker, CCB = calcium—channel blocker.

Fig 3 provided a classification of the prescriptions by type antihypertensive medication among the patients with antihypertensive medications therapy. 55.76% patients with hypertension were prescribed an antihypertensive medication, the most prescribed were calcium channel blockers. Urban participants, compared with rural participants, more frequently used a combination of antihypertensive drugs, most were single pill combination treatment (*P*<0.001, Fig 3).

### Factors associated with hypertension on prevalence, awareness, treatment and control

Logistic regression models including crude model and adjusted model were used to assess factors associated with hypertension on prevalence, awareness, treatment and control, respectively. The model adjusted for demographic factors such as gender, region, age, education level, retired status, marital status, BMI, family history of hypertension, showed that elderly, male, retired, single, living in rural areas compared with living in urban areas, low level of education, family history of hypertension, alcohol use, overweight, obesity, central obesity, visceral adipose, high body fat percentage and high FPG were associated with hypertension (Table 3).

**Table 3:** Factors associated with hypertension prevalence among adults and awareness, treatment and control among hypertensive in southern China in 2013.

Variables	hypertension prevalence		Hypertension awareness		Hypertension treatment		Hypertension control	
	Crude OR (95% CI)	Adjusted OR*(95% CI)	Crude OR (95% CI)	Adjusted OR*(95% CI)	Crude OR (95% CI)	Adjusted OR*(95% CI)	Crude OR (95% CI)	Adjusted OR*(95% CI)
Age (ref: 15–39 years)								
40–49	5.0 (4.3–5.7)	4.0 (3.4–4.7)	4.5 (3.3–6.1)	4.3 (3.1–6.0)	6.3(4.2–9.5)	5.8 (3.9–8.8)	4.9 (3.0–7.9)	5.6 (3.4–9.3)
50–59	12.6 (10.9–14.4)	10.5 (8.9–12.4)	7.8 (5.8–10.5)	7.8 (5.7–10.8)	11.2 (7.5–16.5)	10.6(7.1–15.8)	7.3 (4.6–11.6)	10.2 (6.3–10.7)
60 and above	32.8 (29.0–37.0)	32.4 (27.5–38.2)	11.2 (8.5–14.8)	11.5 (8.4–15.7)	17.7 (12.1–25.8)	18.1 (12.2–26.7)	7.6 (4.9–11.9)	13.4 (8.3–21.7)
Gender(ref: Female)	1.2 (1.1–1.2)	1.4 (1.3–1.6)	0.6 (0.5–0.7)	0.7 (0.6–0.8)	0.6 (0.5–0.7)	0.7 (0.7–0.8)	0.9 (0.8–1.0)	0.8 (0.7–0.9)
Retired(ref: No)	3.9 (3.6–4.2)	1.2 (1.1–1.4)	1.9 (1.7–2.1)	1.2 (1.0–1.4)	1.8 (1.6–2.0)	1.1 (0.9–1.2)	1.3 (1.1–1.4)	1.0 (0.9–1.2)
Region(ref: Urban)	1.1 (1.0–1.1)	1.1 (1.0–1.2)	0.8 (0.7–0.9)	0.8 (0.7–0.9)	0.8 (0.7–0.9)	0.8 (0.7–0.9)	0.7 (0.6–0.7)	0.7 (0.6–0.8)
Marital (ref: Married)	0.6 (0.6–0.7)	1.3 (1.2–1.5)	0.9 (0.8–1.1)	0.8 (0.7–0.9)	1.0 (0.9–1.2)	0.9 (0.7–1.0)	0.7 (0.6–0.8)	0.8 (0.7–1.0)
Education level (ref: College or higher)								
Middle	2.0 (1.7–2.3)	1.1 (0.9–1.4)	0.9 (0.7–1.2)	1.7 (1.5–2.0)	0.7(0.6–0.9)	1.5 (1.4–1.8)	0.6 (0.5–0.9)	0.5 (0.4–0.7)
Primary	7.2 (6.2–8.4)	1.2 (1.0–1.5)	1.4 (1.0–1.9)	1.6 (1.4–1.9)	1.1 (0.8–1.4)	1.4 (1.3–1.6)	0.7 (0.5–0.9)	0.5 (0.3–0.6)
Illiterate	1.2 (1.0–1.4)	1.4 (1.2–1.8)	1.5 (1.1–2.0)	1.5 (1.4–1.8)	1.1 (0.8–1.5)	1.3 (1.2–1.5)	0.5 (0.4–0.7)	0.3 (0.2–0.4)
BMI (ref: Normal)								
Overweight	2.5 (2.4–2.7)	2.4 (2.2–2.6)	1.4 (1.2–1.6)	1.7 (1.4–1.9)	1.3 (1.2–1.5)	1.6 (1.4–1.8)	1.0 (0.9–1.2)	1.1 (0.9–1.2)
Obese	4.2 (3.8–4.7)	5.7 (4.9–6.5)	1.2 (1.0–1.5)	1.8 (1.5–2.3)	1.1 (0.9–1.3)	1.7 (1.4–2.0)	0.8 (0.7–1.0)	1.0 (0.8–1.2)
AWC(ref:<90M,<85 F)								
≥90 for M,≥85 for F	3.1 (2.8–3.4)	1.7 (1.5–2.0)	1.4 (1.2–1.6)	1.6 (1.3–1.9)	1.3 (1.1–1.5)	1.2 (1.0–1.4)	1.1 (0.9–1.3)	1.2 (1.0–1.5)
≥95 for M,≥90 for F	4.6 (4.2–5.1)	1.8 (1.5–2.1)	1.5 (1.3–1.7)	1.6 (1.4–1.9)	1.4 (1.2–1.6)	1.1 (0.9–1.4)	0.9 (0.8–1.1)	1.0 (0.8–1.2)
FHH (ref: No)	1.2 (1.1–1.2)	2.0 (1.8–2.2)	1.3 (1.1–1.5)	2.4 (2.0–2.8)	1.1 (1.0–1.3)	2.1 (1.8–2.5)	1.3 (1.1–1.5)	1.6 (1.3–1.8)
Smoker (ref: No)	1.2 (1.1–1.3)	1.0 (0.9–1.1)	0.6 (0.5–0.7)	0.8 (0.7–0.9)	0.6 (0.5–0.6)	0.8 (0.6–0.9)	0.7 (0.6–0.8)	0.7 (0.6–0.9)
Drinker (ref: No)	1.6 (1.5–1.7)	1.3 (1.1–1.4)	0.7 (0.6–0.8)	0.9 (0.8–1.1)	0.7 (0.6–0.8)	0.8 (0.7–0.9)	0.8 (0.7–0.9)	0.8 (0.6–0.9)
Excessive salt use(ref: <6 gram)	1.3 (1.2–1.4)	0.9 (0.9–1.0)	1.0 (0.9–1.1)	0.9 (0.8–1.0)	1.0 (0.9–1.1)	0.8 (0.7–0.9)	1.0 (0.9–1.1)	1.0 (0.9–1.1)
Diabetes (ref: No)	1.7 (0.9–3.5)	1.6 (0.8–3.5)	0.8 (0.3–2.6)	0.5 (0.1–1.7)	1.3 (0.5–3.4)	0.8 (0.2–2.4)	1.1 (0.4–3.2)	0.8 (0.2–2.6)
TG (ref: <2.26)	2.5 (2.1–3.0)	1.4 (1.2–1.8)	0.7 (0.5–0.9)	0.7 (0.5–0.9)	0.7 (0.5–0.9)	0.6 (0.4–0.8)	0.5 (0.4–0.7)	0.5 (0.4–0.8)
TC (ref: <6.22)	2.5 (2.0–3.0)	1.1 (0.9–1.4)	1.0 (0.7–1.3)	0.8 (0.6–1.1)	0.7 (0.5–0.9)	0.5 (0.4–0.8)	0.5 (0.4–0.8)	0.5 (0.3–0.8)
FBG (ref: ≤6)								
6.1–6.9	3.7 (2.9–4.7)	1.7 (1.3–2.3)	1.2 (0.8–1.8)	1.0 (0.7–1.5)	1.0 (0.7–1.4)	0.8 (0.6–1.1)	1.0 (0.7–1.4)	0.8 (0.6–1.2)
≥7	4.4 (3.3–5.8)	1.9 (1.3–2.6)	1.0 (0.7–1.5)	0.9 (0.6–1.3)	0.9 (0.6–1.3)	0.7 (0.5–1.1)	0.7 (0.4–1.1)	0.7 (0.4–1.1)
VAI(ref: <10)								
10–14	3.5 (3.2–3.8)	1.5 (1.4–1.7)	1.2 (1.0–1.3)	1.4 (1.2–1.7)	1.1 (0.9–1.2)	1.1 (0.9–1.3)	1.0 (0.9–1.1)	1.0 (0.8–1.2)
15–30	5.7 (5.1–6.5)	2.0 (1.6–2.4)	1.6 (1.3–1.9)	2.1 (1.7–2.6)	1.4 (1.2–1.7)	1.4 (1.1–1.9)	1.0 (0.8–1.2)	1.0 (0.7–1.3)
BFP (ref: <10 for M, <20 for F								
10–19 for M,20–29 for F	0.6 (0.5–0.7)	0.6 (0.5–0.8)	0.6 (0.4–0.8)	0.6 (0.4–0.8)	0.5 (0.4–0.7)	0.7 (0.5–0.9)	1.1 (0.8–1.6)	1.1 (0.8–1.6)
20–24 for M 30–34 for F	2.1 (1.8–2.4)	1.0 (0.8–1.3)	0.7 (0.5–1.0)	0.7 (0.5–1.0)	0.7 (0.6–0.9)	0.8 (0.6–1.1)	1.1 (0.8–1.5)	0.9 (0.7–1.3)
≥25 for M, ≥35 for F	5.0 (4.3–5.8)	1.3 (1.0–1.5)	1.2 (0.9–1.6)	1.1 (0.8–1.5)	1.1 (0.8–1.4)	0.9 (0.7–1.2)	1.2 (0.9–1.7)	1.2 (1.0–1.4)

FHH: Family history of hypertension. BMI: Body mass index. TG: Triglycerides. TC: Total cholesterol. HDL-C: high density lipoprotein cholesterol. FBG: Fasting blood glucose. BFP: Body fat percentage. M: Male. F: Female. AWC: Abdominal waist circumference. BMI: Body mass index.

*Adjusted for gender, region, age, education level, retired status, marital status, BMI, Family history of hypertension.

It showed that among those with hypertension, elderly, female, retired, living in urban, married, level of education, overweight, obesity, central obesity, family history of hypertension, nonsmoking, visceral adipose were associated with hypertension awareness (Table 3).

In the adjusted model, among those with hypertension, elderly, female, living in urban places, level of education, overweight, obesity, family history of hypertension, nonsmoking, nondrinking, low salt taken, low TG, TC and high visceral adipose index were associated with hypertension treatment (Table 3). Meanwhile, elderly, female, living in urban areas, high level of education, family history of hypertension, nonsmoking, nondrinking, low TC, low TG and high body fat percentage affecting control of hypertension (Table 3).

## Discussion

Hypertension has been identified as one of the leading risk factors for global burden of disease and the main risk factor for mortality [13–15]. The prevalence of hypertension among the adult population globally is predicted to increase from 26% in 2000 to 29% by the year of 2025 [16,17]. According to our survey, the prevalence of hypertension in Zhejiang province is 24.56%, increasing more than 19.5%, compared with the prevalence of 19.8% in 2002 [2]. It is lower than those in northern parts of China and higher than developed countries such as United States [18–20]. The prevalence of pre-hypertension is 32.11%, with the most prevalent subgroup of 40–49 which is also the most important workforce group that means more than one third of Zhejiang adult population would have hypertension or elevating risk of CVD for the next few years, if we don’t take any effective measures [21–23].

Awareness, treatment and control of hypertension among adults in Zhejiang province in 2013 were 67.43%, 55.76% and 30.79% respectively, all increased significantly compared with 2002. The improvement was likely to be due in part of the national campaign of hypertension prevention and control proposed by government, as well, people have paid more attention to their health condition with the rapid development of China’s economy. Although hypertension awareness, treatment and control have markedly improved, there were still gaps compared with those of developed countries [20,24,25]. Control of hypertension was unsatisfactory, especially among young and middle-aged population, which remained only 5.85% and 24.87% for 15–39 and 40–49 years old subgroups respectively, and the awareness, treatment were also relative low for them. That might mainly be caused by the neglect and their busy work lifestyle habits including of more stress, with the rapid urbanization in the past few decades in China, which resulted in uncontrolled hypertension. More effective primary prevention measures should be made for the young and middle-aged population, not just for the elderly, to address the rise in hypertension and pre-hypertension.

In our survey, those using a combination of antihypertensive drugs including single pill combination treatment had higher control rate compared with those using mono therapy (60.17% vs 52.94%, *P*<0.0001). More patients used inappropriate antihypertensive medications in rural areas compared with those in urban areas, probablely due to the lack of qualified physicians in these areas.

Gender, age, BMI, high TG, high FPG, living in rural areas, low level of education, and family history of hypertension are traditional associated factors of hypertension [1,3,26–28], our study provide additional support to these hypothesis. Some reports have shown that excessive alcohol use and tobacco consumption are also important causes of hypertension [3,29,30], our results support for the alcohol hypothesis, but not for the latter one. In our study, visceral adipose and high body fat percentage were showed also associated with hypertension, the SBP and DBP gradually increased with VAI and BFP. Our study also showed that VAI might have a more important effect on SBP/DBP for women, compared with men. BFP might also have more important effect on DBP for women. VAI/BFP might have a more important effect on SBP/DBP for rural areas, compared with urban areas. Some studies showed that overweight, obesity or central obesity was significantly associated with hypertension [2,3,31–32], our results further confirmed these studies. It is likely that overweight, obesity and central obesity in southern Chinese adults are major contributing factors of hypertension.

Our survey showed hypertension awareness and treatment was higher for female which was observed either in other areas [33–35], and the difference maybe because of the developmental and personality factors [3,36]. The hypertension awareness and treatment increased with age, and patients living in urban places, overweight, obesity, with family history of hypertension and higher VAI had higher awareness and treatment rates.

Control of hypertension was higher for female, and patients living in urban places, having higher level of education, having better lifestyles such as nonsmoking or nondrinking had better control of hypertension, which were similar with some other studies [1,31,35]. In our survey, BFP more than 25 for men and 35 for women was associated with uncontrolled hypertension.

Our study had several strengths and limitations. The main strength of this study was the large sample size, coverage, representativeness of the region population, which were selected according to the national demographic by gender and age. Moreover, it provided new information about hypertension in the general adult population of Southern China. Despite these strengths, there were several limitations. First, our study was a cross-sectional survey, which was failed to establish cause-and-effect relationship between the observed associations. Second, some confounding variables including dietary habits and family income were not included.

In conclusion, our results showed an increasing prevalence of hypertension and high pre-hypertension in the general population in southern China, but levels of awareness, treatment, and control remain relatively low, especially for young and middle-aged population. Faced with the epidemiological transition, we need innovative strategies to control and prevent hypertension, including modifying risk factors such as high weight, inappropriate use of antihypertensive drugs and conducting community-based intervention programs to address this serious problem.

## Supporting Information

S1 Table Minimal data set. (XLS)

S2 Table Healthy condition questionnaire. (DOCX)
