# Supplementary material for: Prevalence, Awareness, Treatment, Control and Risk Factors Associated with Hypertension among Adults in Southern China, 2013
Source: PLoS One. 2016 Jan 19;11(1):e0146181. doi: 10.1371/journal.pone.0146181 (PMC4718602; doi:10.1371/journal.pone.0146181)
Supplement: S2 Table — (DOCX) [file pone.0146181.s002.docx]

**S2 Table. Healthy condition questionnaire**

Part 1. Behavior and Life style

1. Smoking

B1.1 Have you smoked over last 30 days?

0=Never (turn to B1.3), 1=Yes, smoking everyday, 2=Yes, but not everyday.

B1.1.1 How long have you been having the habit of smoking everyday?

1= < 3 months, 2= 3 to 6 months, 3= 6-12 months, 4=more than 1 year ( _years).

B1.2 How many cigarettes do you usually have? (“999” is for can't remember clearly)

Frequency* Quantity

B1.2.1 Cigarettes made by machine _______ ______

B1.2.2 Handmade cigarettes _______ ______

B1.2.3 Cigarettes smoking with [pipe](javascript:void(0);) _______ ______

B1.2.4 Cigar _______ ______

B1.2.5 Others _______ ______

Note: Frequency *: 0=Never, 1=Yes, smoking everyday, 2=Yes, but not everyday.

B1.3 Did you use to smoke in the past?

0=Never (turn to B1.8), 1=Yes, smoking everyday, 2=Yes, but not everyday.

B1.4 What was your age when you started smoking? __ years old.

B1.5 How long have you been smoking? __ years old.

B1.6 How many cigarettes have you had since you started smoking?

1= <20 cigarettes 2=20 to 400 cigarettes 3= >400 cigarettes.

B1.7 Have you been tried to stop smoking? 0=Never 1=Yes

B1.7.1 How many years since you stopped smoking? ___ years

B1.7.2 What’s the reason for stopping smoking? 1=Disease 2=Others: ________.

B1.8 How many days have you been expose to second-hand smoke usually?

0=Never (turn to B2.1) 1=Yes, ___ days

B1.8.1 How many days have you had that exposure to second-hand smoke over 15 minutes/day?

0=Never (turn to B2.1) 1=Yes, ___ days

1. Drinking

B2.1 Have you ever had wine? 0=Never (turn to B3.1) 1=Yes

B2.2 How old were you when you stared to drink regularly? ___ years.

B2.3 Which one do your drinking habits belong to?

1= More than once everyday. 2= More than once every week. 3= More than once every month. 4= Seasonal drink, drinking __ months every year, __ times every month. 5= Occasional drink, __ times every year.

B2.4 Have you taken alcohol at least once this month? 0= No 1= Yes

B2.5 Do you usually get drunk when you take alcohol?

1= Almost every time 2=  Most of the time 3= Half of the time 4= Part of the time 5= A few time 6= Never

B2.6 Have you been tried to stop drinking? 0=Never 1=Yes

B2.6.1 How old were you when you tried to stop drinking? __ yeras

B2.6.2 How many years since you stopped drinking? ___ years

B2.6.3 What’s the reason for stopping drinking? 1=Disease 2=Others: ______.

B2.7 Drinking condition:

Frequency*, Times**, Quantity, Months/year, Alcohol volume

B2.7.1 Beer ____ ____ ____ ____ _____

B2.7.2 Chinese liquor ____ ____ ____ ____ _____

B2.7.3 Wine ____ ____ ____ ____ _____

B2.7.4 Rice wine ____ ____ ____ ____ _____

B2.7.5 Others ____ ____ ____ ____ _____

Note: Frequency *: 0=Never, 1=Day, 2=Week, 3=Month, 4=Year.

Times**: Which corresponds to Frequency.

1. Diet (Condition of the past year)

B3.1 Food Frequency* Quantity

B3.1.1 Rice, flour, cereal _______ ___ Jin ___ Liang

B3.1.2 Potato _______ ___ Jin ___ Liang

B3.1.3 Vegetable _______ ___ Jin ___ Liang

B3.1.4 Livestock meat _______ ___ Jin ___ Liang

B3.1.5 Poultry _______ ___ Jin ___ Liang

B3.1.6 Fish _______ ___ Jin ___ Liang

B3.1.7 Shellfish _______ ___ Jin ___ Liang

B3.1.8 Eggs _______ ___ Jin ___ Liang

B3.1.9 Milk _______ ___ Jin ___ Liang

B3.1.10 Bean products _______ ___ Jin ___ Liang

B3.1.11 Fruit _______ ___ Jin ___ Liang

B3.1.12 Nuts  _______ ___ Jin ___ Liang

B3.1.13 Pickles _______ ___ Jin ___ Liang

Note: Frequency *: 0=Never, 1=Day, 2=Week, 3=Month, 4=Year.

B3.2 How many times did you have meals at the following places?

B3.2.1 Breakfast 1= Home 2= Canteens 3= Restaurant

B3.2.2 Lunch 1= Home 2= Canteens 3= Restaurant

B3.2.3 Supper 1= Home 2= Canteens 3= Restaurant

B3.3 How many people does your family have meals usually? __

B3.4 How many people that aged less than 6 among the family dinner? __

B3.5 How much [vegetable](javascript:void(0);) [oil](javascript:void(0);) dose your family use a month usually? __ Jin/Month

B3.6 How much animal oil dose your family use a month usually? __ Jin/Month

B3.7 How much sodium salt dose your family use a month usually? __ Jin/Month
